# Supplementing Northern Australian Beef Cattle with *Desmanthus* Tropical Legume Reduces In-Vivo Methane Emissions

**DOI:** 10.3390/ani10112097

**Published:** 2020-11-11

**Authors:** Bénédicte Suybeng, Edward Charmley, Christopher P. Gardiner, Bunmi S. Malau-Aduli, Aduli E.O. Malau-Aduli

**Affiliations:** 1 Animal Genetics and Nutrition, Veterinary Sciences Discipline, College of Public Health, Medical and Veterinary Sciences, Division of Tropical Health and Medicine, James Cook University, Townsville, QLD 4811, Australia; benedicte.suybeng@my.jcu.edu.au (B.S.); christopher.gardiner@jcu.edu.au (C.P.G.); 2 CSIRO Agriculture and Food, Private Mail Bag Aitkenvale, Australian Tropical Sciences and Innovation Precinct, James Cook University, Townsville, QLD 4811, Australia; ed.charmley@csiro.au; 3 College of Medicine and Dentistry, Division of Tropical Health and Medicine, James Cook University, Townsville, QLD 4811, Australia; bunmi.malauaduli@jcu.edu.au

**Keywords:** methane emission, mitigation, tannins, tropical beef cattle, *Desmanthus leptophyllus*, *Desmanthus bicornutus*, phenolics, legumes, polyethylene glycol, greenhouse gas

## Abstract

**Simple Summary:**

The problem addressed in this study is that of mitigating methane emissions by tropical beef cattle with the aim of reducing the impact of climate change and greenhouse gas emissions in Northern Australia. The primary objective was supplementing tropical beef cattle on poor quality hay with incremental levels of *Desmanthus leptophyllus* cv. JCU1 and *Desmanthus bicornutus* cv. JCU4 to evaluate their in-vivo antimethanogenic effect. Results showed that, irrespective of cultivar, incremental supplementation with up to 31% of *Desmanthus* led to a 10% linear decrease in methane emissions without reducing dry matter intake. This finding makes a significant novel contribution to a better understanding of the impact of supplementing beef cattle with *Desmanthus* on in vivo methane reduction and the role of condensed tannins in rumen fermentation. The practical implication of this finding is that *Desmanthus*, an adapted tropical legume, has the potential to mitigate in vivo methane emissions by beef cattle in the drier parts of Northern Australia and contribute to the larger global effort of reducing the impact of climate change and greenhouse gas emission.

**Abstract:**

The main objective of this study was to investigate the effect of supplementing beef cattle with incremental levels of *Desmanthus leptophyllus* cv. JCU1 and *Desmanthus bicornutus* cv. JCU4 on in vivo methane (CH_4_) emissions and the role of tannins in rumen fermentation. Fourteen yearling Droughtmaster steers were allocated to each of the two *Desmanthus* species and offered a basal diet of Rhodes grass (*Chloris gayana*) hay plus fresh *Desmanthus* at 0%, 15%, 22%, and 31% of dry matter intake (DMI). The 15% and 31% *Desmanthus* periods lasted 21 days and the 22 and 0% *Desmanthus* periods, 14 days. Methane production was measured by open-circuit gas exchange in the last two days of each period. The results showed a linear increase in DMI and reduction in CH_4_ yield with the increasing level of *Desmanthus* and subsequently condensed tannins in the diet. The added tannin binder polyethylene glycol-4000 did not affect CH_4_ yield but increased rumen NH_3_-N and iso-acid concentrations. Therefore, on a low-quality diet, *Desmanthus* has the potential to increase intake and reduce CH_4_ emissions. Even though its tannins can bind rumen proteins, the beef cattle anti-methanogenic response to supplementation with *Desmanthus* may be a combination of rumen fermentation and tannin effects.

## 1. Introduction

Agriculture accounted for 14% of Australia’s greenhouse gas (GHG) emissions with enteric methane (CH_4_) fermentation contributing up to 10% of its GHG in 2018 [1]. The state of Queensland has 12 million cattle which accounted for 47.2% of Australian beef and veal production in 2018–2019 [2]. GHG is the principal source of global climate change [3]. Therefore, mitigating the CH_4_ produced by the cattle industry especially in Northern Australia, would offer an opportunity to reduce the impact of GHG emissions and climate change. Approximately half of Australia’s beef cattle population is found in Northern Australia, which is characterized by a tropical and arid climate with rainfall occurring mainly during the wet season of November to April. The extensive grazing system in Northern Australia is characterized by low animal productivity due to poor quality native pastures with low digestibility and comparatively higher CH_4_ emissions than the intensive system [4,5].

A survey focusing mainly on eight sites in semiarid clay soil regions of central-western, north, and north-western Queensland (Blackall, Barcaldine, Longreach, Julia Creek, Isisford, Yaraka, Chillagoe, and Townsville) showed that only some *Desmanthus* accessions survived and thrived among other legumes (*Stylosanthes*, *Alysicarpus*, *Centrosema*, *Chamaecrista*, *Clitoria* and *Vigna*) under harsh conditions (grazing, floods, fires, frost, droughts, and insect attacks) after two decades [6]. The selection and breeding of the surviving plants from these abandoned sites have led to the development of five new cultivars of *Desmanthus* for Northern Australia and similar environments: JCU1 (*Desmanthus leptophyllus*), JCU2 and JCU3 (*D. virgatus*), JCU4 (*D. bicornutus*), and JCU5 (*D. virgatus*) [6]. Some of these cultivars have also shown promising results for reducing in vitro CH_4_ emission and improving animal growth performance [7]. Vandermeulen et al. [8] demonstrated higher anti-methanogenic potential of *D. leptophyllus* cv. JCU1 and *D. bicornutus* cv. JCU4 after 72 h of in vitro rumen fermentation using rumen fluid from Brahman steers compared to a combination of Rhodes grass hay and *D. virgatus* cv. JCU2. Their study showed a 21% CH_4_ reduction with JCU1, 26% with JCU4 and 5% with JCU2 when sampled in March after 51 days of regrowth, compared to Rhodes grass [8]. Durmic et al. [9] showed a potential 48% and 45% in vitro CH_4_ mitigation in summer with JCU1 and JCU4 respectively, using sheep rumen fluid. They postulated that the observed CH_4_ reduction may be caused by secondary compounds in *Desmanthus,* such as condensed tannins (CT), hydrolysable tannins (HT), and total phenolics (TP) [8,10,11]. Both *D. leptophyllus* and *D. bicornutus* are erect shrubs (0.4–3 m tall). *D. leptophyllus* is woody at the base and usually much branched whereas *D. bicornutus* is unbranched or occasionally 2–3 branches from the base and becomes woody at the base with age [12]. The crude protein (CP) content of *D. leptophyllus* ranges between 10.5% to 15.5% and 15% to 27% for *D. bicornutus* [13]. JCU1 was selected on the basis of its persistence under grazing and plant density relative to known *Desmanthus* cultivars [14]. JCU4 is a robust early maturing plant compared to JCU1 which is late maturing (84 and 95 days for the first flowering from sowing respectively) [6,14]. Furthermore, previous studies with steers [15,16], sheep [17,18], and goats [19] supplemented with *Desmanthus* showed significant liveweight (LW) gains. *Desmanthus* has the potential to be a promising legume for animal growth and CH_4_ reduction. However, to our current knowledge, no study has been conducted to explore the in vivo CH_4_ mitigation capability of *Desmanthus* as a supplement in tropical beef cattle on poor quality feeds. This represents a major knowledge gap that the present study intended to fill.

Therefore, the primary objective of this study was to investigate the effect of supplementing beef cattle with incremental levels of JCU1 and JCU4 (which showed a higher anti-methanogenic potential compared with JCU2 in vitro [8]) on in vivo CH_4_ emissions, LW gain and rumen metabolites. The hypothesis tested was that feeding tropical steers with incremental levels of JCU1 and JCU4 will decrease CH_4_ emissions due to the presence of tannins without negatively affecting rumen metabolites and increase the animals’ LW gain.

## 2. Materials and Methods

This study was conducted at the Commonwealth Industrial and Scientific Research Organisation (CSIRO) Lansdown Research Station, Queensland, Australia (19.59° S, 146.84° E) following the Australian Code for the Care and use of Animals for Scientific Purposes (eight edition, 2013) and was approved by the CSIRO Queensland Animal Ethics Committee (permit A02/2018).

### 2.1. Animals and Treatments

Fourteen yearling Droughtmaster steers with an average LW of 296 ± 5 kg were blocked by weight and randomly allocated to three groups of 4 animals each, and one group of 2 animals. Half of the animals in each group were allocated to either *D. leptophyllus* cv. JCU1 or *D. bicornutus* cv. JCU4. Rhodes grass (*C. gayana*) hay was offered to the animals and 4 proportions of *Desmanthus* were offered to the animals as follows: 15%, 31%, 22%, and 0% of dry matter (DM). The 15% and 31% of *Desmanthus* DM periods lasted 21 days and the last two periods (22% and 0% *Desmanthus* DM) lasted 14 days due to time constraints. The adaptation period to the experimental diet was within the 10–14-day range suggested by Cochran and Galyean [20] and considered adequate. During the 22% *Desmanthus* period, 6 animals (3 animals on each cultivar at 22% *Desmanthus*) were supplemented with polyethylene glycol (PEG, MW 4000, Chem-Supply Pty Ltd., Gillman, SA, Australia) at 160 g/kg *Desmanthus* DM to nullify the bioactivity of tannins. Although consumption problems with PEG supplement were not anticipated, the animals were nonetheless fed an increasing amount of PEG (50 g/day) for 5 days before reaching their full amount prior to the experimental period for adaptation purposes. All animals were fed ad libitum to 10% refusals over the first seven days of each period. Thereafter, intake was reduced to 90% of ad libitum. Methane production was measured by open-circuit gas exchange in the last 2 days of each period. Both *Desmanthus* cultivars were harvested fresh using a crop chopper (New Holland Model 38 Crop-Chopper^®^, Haryana, India) on alternate days from a farm located 20 min away from the research station (19.67° S, 146.96° E). The fresh *Desmanthus* was consistently harvested at 8:30 between four and six weeks of regrowth to capture the vegetative stage of maturity to minimize differences in nutritive value between the cultivars. The *Desmanthus* was stored at 5 °C in a cool room prior to feeding. Immediately before feeding, *Desmanthus* was mixed with chopped (Roto grind model 760, Burrows Enterprises, LLC, Greeley, CO, USA) Rhodes grass hay. Diets were fed once daily at 9:30–10:00 and all experimental steers had continuous access to reticulated water and mineral block (Trace element Northern, Olsson’s, Yennora, NSW, Australia).

### 2.2. Feed Chemical Composition and Analysis

The DM content of the basal and experimental diets was determined by drying samples to a constant temperature at 60 °C in a fan forced oven for 48 h. The DM was calculated as the difference between the initial and final weights of samples expressed as a percentage. The oven dried samples were ground to pass through a 1-mm screen using a Tecator Cyclotec 1093 (FOSS, Hillerød, North Zealand, Denmark) for neutral (NDF) and acid detergent fiber (ADF) and total nitrogen analysis. Concentrations of NDF and ADF were measured sequentially using the filter bag method from the operating instructions of the ANKOM 200/220 Fiber Analyzer (ANKOM Technology, Fairport, NY, USA). The analysis for total nitrogen was determined by combustion using a Leco CN628 N Analyser (Leco, St. Joseph, MI, USA) [21] and the values multiplied by 6.25 to give the CP percentage. In vitro DM digestibility (DMD) was determined using a modified pepsin-cellulase technique described by Clarke et al. [22]. Metabolizable energy (ME) was calculated from in vitro true digestibility as DMD x 0.172–1.707 [23]. Crude protein intake was calculated as the CP of the dry feed offered minus the CP of the dry feed refused after 24 h.

### 2.3. Extraction and Analyses of Condensed Tannins and Total Phenolics

Both *Desmanthus* cultivars were freshly sampled every week. The samples were stored at −20 °C, then freeze-dried at −50 °C for 3 days in a freeze dryer (Labogene ScanVac CoolSafe freeze dryer, Bjarkesvej 5 DK-3450, Allerød, Denmark) and ground to pass a 1-mm screen using a Tecator Cyclotec 1093 (FOSS, Hillerød, North Zealand, Denmark) and stored at room temperature (20 °C) [24]. The freeze-dried material was passed through a 0.25 mm sieve before analysis. Tannin extraction from the *Desmanthus* samples followed the procedure described by Terrill et al. [24] except that the supernatant was diluted with distilled water to a total volume of 300 µL. Proanthocyanidin concentration (CT) was estimated by the Butanol-HCl-Fe^III^ method using purified *Desmanthus* CT as the standard with absorbance detection at 550 nm [25,26]. Condensed tannins were purified on Sephadex LH-20 as described by Wolfe et al. [27]. Total phenolics concentration was determined by the Folin-Ciocalteu method with catechin as the standard [25].

### 2.4. Dry Matter Intake and Liveweight Gain

The LW of each animal was recorded weekly prior to feeding to determine the daily LW gain. Individual DMI was determined by the difference between offered and residual feed after 24 h. Individual daily intakes were recorded throughout the study to determine treatment group DMI. These values were used to calculate the DMI expressed as % of LW and to express the CH_4_ yield on per kg DMI basis.

### 2.5. Rumen Collection and Volatile Fatty Acids (VFA) Analysis

Rumen fluid samples were collected through an oral stomach tube using a reinforced plastic suction tube (approximately 3 cm in diameter). A hand pump was used to extract 100–200 mL of rumen fluid from the ventral sac. The rumen fluid was collected 3 h post-feeding following the second day of confinement in respiration chambers. pH of the rumen fluid was immediately measured using a pH meter and a sub-sample taken, mixed with fresh 20% metaphosphoric acid (4:1) and frozen at −80 °C for VFA and NH_3_-N analyses. Rumen fluid concentrations of short chain fatty acids (acetate, propionate, n-butyrate, iso-butyrate, iso-valerate, n-valerate, and n-caproate) were measured by gas chromatography(Shimadzu Corporation, Kyoto, Japan as described by Gagen et al. [28]. NH_3_-N concentration was determined by the colorimetric method of Chaney and Marbach [29].

### 2.6. Measurement of CH_4_ Emissions

Four open-circuit respiration chambers were used to assess CH_4_ production from individual steers as described by Martinez-Fernandez et al. [30]. Briefly, CH_4_ emissions were measured using independent units (23.04 m^3^, 3000 L/min airflow) equipped with drinking water and a feed bin containing the daily ration. The atmosphere inside the chambers was maintained at 2 °C below ambient temperature, approximately −10 Pa and a relative humidity between 50% to 75%. The exact flow rates of each chamber were corrected to measured conditions for temperature and pressure to calculate CH_4_ production [31]. Steers remained in the chambers for 48 h with CH_4_ monitored continuously by infrared absorption (Servomex 4100, Servomex Group Ltd. Crowborough, UK). Methane production was calculated by averaging two 24 h measurements. DMI in the chamber was also recorded daily to calculate the CH_4_ emissions according to feed intake (CH_4_ yield expressed as g/kg DMI).

### 2.7. Statistical Analyses

All data were analysed using R (Rstudio version 1.3.1056, R Core Team (2013). R: A language and environment for statistical computing. R Foundation for Statistical Computing, Vienna, Austria, ISBN 3-900051-07-0, URL http://www.R-project.org/) with the ‘dplyr’, ‘nlme’, ‘agricolae’, ‘MuMIn’, ‘car’, ’Metrics’ and ‘multcomp’ packages. Effects were considered significant at *p* < 0.05.

Multiple Analysis of variance (MANOVA) and linear mixed model procedures were conducted to compare the chemical compositions between JCU1 and JCU4 cultivars and their effects in the diet on intake, daily LW gain, CH_4_ yield and products of rumen fermentation. The DM, CP, ADF, NDF, ME, TP, CT, DMI, CP intake, daily LW gain, VFA, pH, and NH_3_-N were the dependent variables, while *Desmanthus* cultivars (JCU1 and JCU4), level of *Desmanthus* in the diet, and PEG were the fixed effects and individual animals nested within treatment groups were the random effects.

A linear mixed model procedure was used to analyze the impact of the percentage of *Desmanthus* in the diet on the nutritive value of the treatments (JCU1 and JCU4), animal production, CH_4_ emissions, and products of rumen fermentation. The same model was also used to examine the impact of DMI, percentage of *Desmanthus* in the diet, CT, TP, and CP on CH_4_ production (g/day) or CH_4_ yield (g/kg DMI). The model was fitted with the restricted maximum likelihood (REML) technique with the DMI, percentage of *Desmanthus* in the diet, percentage of CT, TP or CP as a fixed effect and individual animals nested within treatment groups as random effects. When significant differences were detected, differences among means were tested by pairwise comparisons (Tukey test).

## 3. Results

### 3.1. Chemical Composition

**Figure 1:**
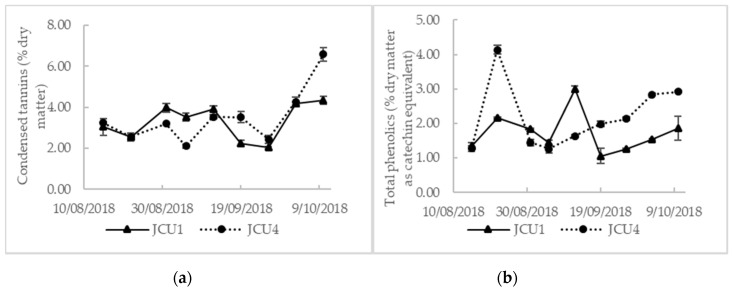
Variation in (**a**) condensed tannins (% dry matter) and (**b**) total phenolics (% dry matter as catechin equivalent) throughout the feeding period.

The composition of Rhodes grass hay is given in Table 1. Rhodes grass had a lower CP concentration than both *Desmanthus* cultivars. Rhodes grass contained less TP than *Desmanthus* and CT was not detected in the Rhodes grass. As displayed in Table 1, the CP, ME, and TP were higher in JCU4 than in JCU1. JCU1 had a higher DM and fiber concentration than JCU4. There was no difference in the concentrations of CT between the two cultivars. The CT and TP in JCU1 and JCU4 were not significantly different throughout the trial (Figure 1).

### 3.2. Cultivar Effects

The higher quality of JCU4 (Table 1), resulted in a significantly higher CP concentration in the diet (Table 2) and subsequently a higher CP intake of animals fed JCU4 compared to the animals fed JCU1 (0.36 ± 0.025 and 0.45 ± 0.033 kg/day for JCU1 and JCU4 respectively). The lower ADF concentration in JCU4 (Table 1) significantly reduced ADF concentration in the diet with JCU4 compared to JCU1 (Table 2). However, there was no significant difference between cultivars for DMI, daily LW gain, CH_4_ yield, and products of rumen fermentation, except for the rumen concentration of iso-valerate (0.56 ± 0.036 and 0.67 ± 0.034 molar % for JCU1 and JCU4 respectively) and n-valerate (0.66 ± 0.018 and 0.76 ± 0.022 molar % for JCU1 and JCU4 respectively).

### 3.3. Desmanthus Level Effects

The DMI, DMI per kg of LW, CH_4_ production and CH_4_ yield followed a linear increase pattern with an increase in the percentage of *Desmanthus* in the diet (Table 3). Methane yield decreased with an increase in *Desmanthus* in the diet (Figure 3a). Every 10% increase in *Desmanthus* intake decreased CH_4_ yield by 3.3%. The addition of PEG to the 22% *Desmanthus* treatments had no influence on CH_4_ yield, intake, daily LW gain and VFA except for an increase in iso-butyrate (0.61 ± 0.0282 and 0.44 ± 0.0355 molar % with and without PEG respectively) and iso-valerate (0.75 ± 0.0449 and 0.51 ± 0.0525 molar % with and without PEG respectively). PEG also significantly increased the concentration of NH_3_-N (12.8 ± 1.85 and 7.7 ± 1.29 mg/dL with and without PEG respectively). The daily LW gain was not correlated to the percentage of *Desmanthus* in the diet.

**Figure 2:**
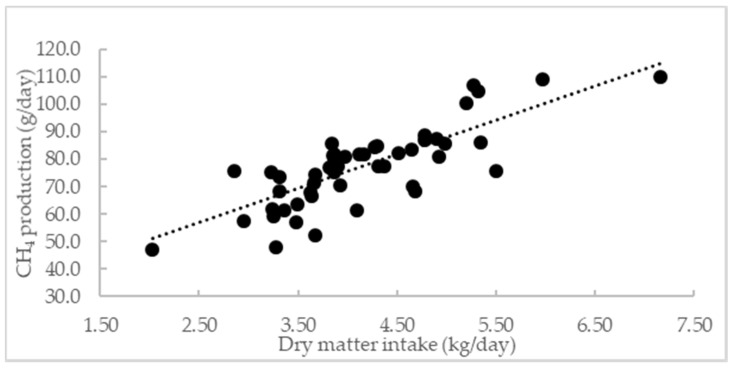
The relationship between dry matter intake (kg /day) and CH_4_ production (g/day). The relationship can be described as CH_4_ production (g/day) = 26.11 + 12.39X, where X = dry matter intake (kg/day) R^2^ = 0.74, *p* < 0.0001.

**Figure 3:**
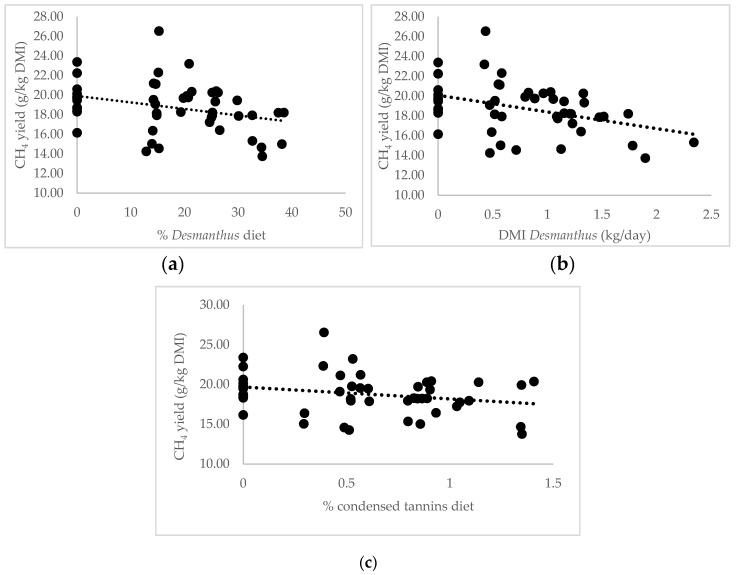
Relationship between CH_4_ yield (g/kg DMI) (y) and (**a**) percentage of *Desmanthus* in the diet (y = 19.92 − 0.066X, where X = percentage of *Desmanthus* in the diet, R^2^ = 0.20, *p* = 0.0097), (**b**) *Desmanthus* dry matter intake (kg/day) (y = 20.08 − 1.68X, where X = *Desmanthus* dry matter intake, *R*^2^ = 0.25, *p* = 0.00075), (**c**) percentage of condensed tannins in the diet (y = 19.67 − 1.49X, where X = percentage of condensed tannins in the diet, R^2^ = 0.15, *p* = 0.035).

Dry matter intake was highly correlated to CH_4_ production (R^2^ = 0.74) (Figure 2). One kg increase in DMI per day increased CH_4_ production by 47%. Methane yield followed a linear regression with the increase in the *Desmanthus* percentage in the diet (Figure 3). Thirty percent of *Desmanthus* in the diet decreased CH_4_ yield by 10%. The increase in *Desmanthus* DMI and CT in the diet also induced a linear decrease in CH4 yield. One kg of *Desmanthus* and 1% of CT in the diet decreased CH_4_ yield by 8%. However, no correlation was found between CH_4_ yield and the percentage of TP in the diet (*p* = 0.22).

The concentration of total VFA, acetate, acetate/propionate ratio, n-valerate, and NH_3_-N significantly increased with an increase in the percentage of *Desmanthus* in the diet (Table 4). On the other hand, the concentration of propionate, iso-butyrate, and iso-valerate decreased with the percentage of *Desmanthus* in the diet. There was no correlation between the level of n-butyrate, n-caproate, and the rumen pH with the increasing level of *Desmanthus* in the diet.

## 4. Discussion

### 4.1. Chemical Composition

*D. bicornutus* (JCU4) is an early maturing suffruticosa plant [6] compared to *D. leptophyllus* (JCU1) [12]. The quality variation between JCU1 and JCU4 might also be due to species differences where the CP in the current trial averaged 11.0% and 14.6% for JCU1 and JCU4, respectively. Cook et al. [13] found that the CP of *D. leptophyllus* ranged from 10.5% to 15.5% compared to 15% to 27% for *D. bicornutus*. Despite their chemical compositional differences, JCU1 and JCU4 had a similar CT concentration (3.5% and 3.7%, respectively), although the TP concentration of JCU4 (2.3% as catechin equivalent) was significantly higher than that of JCU1 (1.7% as catechin equivalent). Vandermeulen et al. [8] reported similar CT concentrations of 3.7% and 4.0% (expressed as leucocyanidin equivalent) for JCU1 and JCU4, respectively. They also reported a higher TP concentration in JCU1 (8.7% as tannic acid equivalent) than in JCU4 (7.3% as tannic acid equivalent). Gonzalez et al. [32] demonstrated that there was a lower concentration of tannins in stems than leaves. This could explain the lower TP concentration in JCU1 which was more mature than JCU4 with fewer leaves. Naumann et al. [10] reported a CT concentration of 8.1% in *D. illinoensis* using a species-specific standard compared to the finding of Gonzalez et al. [32] who reported a tannin concentration of 1.7% in *D. illinoensis* using a vanillin-HCl method and catechin equivalent [33]. The latter found a tannin concentration of 2.1% as catechin equivalent for *D. virgatus* which was lower than the 8.9% catechin equivalent reported by Ramirez et al. [34] for the same *Desmanthus* species using the Burns [33] method modified by Price et al. [35]. These differences in tannin concentration show that even if species-specific CT as the internal standard is used as the most appropriate option for CT analysis [36,37], the tannin results can only be useful in determining relative differences between *Desmanthus* cultivars throughout the experiment rather than for producing absolute quantitative values. Furthermore, the results can vary between laboratories depending on the standards used, presence or absence of water, impurities, light, temperature and time of color development [27].

### 4.2. Cultivar Effects

The results showed a higher CP intake when the animals were fed JCU4 compared to JCU1, probably due to the higher CP concentration in JCU4 (Table 1). However, no cultivar effect was observed on DMI, daily LW gain, pH, rumen metabolites, and NH_3_-N rumen concentrations. The similar CH_4_ yield results detected between the two cultivars were in agreement with the in vitro study conducted by Vandermeulen et al. [8] where they found that expressed per g of fermented OM, CH_4_ production in JCU1 and JCU4 were not significantly different. Looking at the overall data of the three sampling periods (March, August, and October) studied, Vandermeulen et al. [8] also showed similar in vitro acetate/propionate ratios to our study (4.4) for JCU1 and JCU4 (5.0 and 4.8 respectively). In our study, the concentrations of n-valerate and iso-valerate were significantly higher in the rumen of the animals fed JCU4 than JCU1. As reported by Hristov et al. [38], the concentration of valerate and branched-chain VFA were increased or tended to be increased with the increase of dietary N as branched-chain VFA are derived from branched-chain amino acids [39]. Consequently, the concentration of iso-valerate and n-valerate were higher in the animals fed JCU4 compared to the ones fed JCU1 due to the higher CP intake for the animals fed JCU4.

Therefore, it seems that the differences in chemical composition between the two *Desmanthus* species had no major effects on animal performance, CH_4_ emissions, and rumen function.

### 4.3. Animal Performance

Compared with other studies that showed an increased LW gain with *Desmanthus* in cattle [15,16], sheep [17,18], and goats [19], our results showed a low intake and animal performance due to the poor-quality diet. Restricted CP availability has been descried as the critical threshold for suitable microbial growth on the fibrous carbohydrates in basal forage which induces a decrease in animal performance and intake [40,41]. Detmann et al. [42] estimated the concentration of CP in the diet to the apparent equilibrium point where the efficiency of nitrogen utilization in the animal’s body is nil to be 10.8% DM. Yet, in our study, that level was only reached at 22% and 31% of JCU4 in the diet. Moreover, according to the EDGE manual [43], cattle at 300 kg LW will be at maintenance for a diet with an ME of 7 MJ/kg DMI, and a DMI/kg LW of 1.8%,. Thus, in our study, with a ME of the diet averaging 6.6 MJ/kg DMI and a DMI/kg LW of 1.4%, the low animal performance was expected with a daily LW gain of 0.2 kg/head. Furthermore, the high fiber content of the Rhodes grass hay used (NDF = 76.2% DM) could have depressed the rumen microbial digestion of roughages as Dixon [44] showed that roughages of low N content, high fiber content and low digestibility are likely to be most affected by a depression in rumen microbial digestion.

The low-quality diet also had an impact on the rumen NH_3_-N concentration as the CP concentration in the diet is correlated to the NH_3_-N concentration [45,46]. Ensuring adequate rumen NH_3_-N concentration to supply the majority of N for supporting microbial growth is the first priority in optimizing fermentative digestion of forage [40]. Satter and Slyter [47] suggested the optimal ruminal NH_3_-N concentration for maximal microbial growth to be 5 mg/dL. However, a more recent study conducted by Detmann et al. [42] showed that a rumen NH_3_-N concentration of 6.3 mg/dL was needed to reach the equilibrium efficiency of nitrogen utilization. Although the NH_3_-N concentration 3 h after feeding increased with an increase in *Desmanthus* in the diet, the concentration was just above the optimal concentration at 0% *Desmanthus* in the diet (6.4 mg/dL).

Previous studies considered the presence of tannins in feed as anti-nutritive, due to its negative effects on intake, digestion and absorption of nutrients and subsequently animal performance [48]. Vandermeulen et al. [8] showed a decrease in organic matter digestibility (OMD) in vitro with JCU1 compared to JCU4, possibly due to the lower concentration of HT in JCU4 compared to JCU1. The anti-nutritive properties and toxicity of tannins are frequently attributed to a high HT ingestion due to its poorer protein absorption and release of metabolites in the rumen causing cellular damage [49]. Unpalatability due to astringent tannins can lead to reduction in voluntary feed intake [48]. The optimal tannin concentration level in which intake is reduced has not been definitively determined. For instance, Grainger et al. [50] showed a decrease in intake when cows were fed a CT concentration of 0.86% and 1.5% of DM, while Dschaak et al. [51] showed a decrease in intake with a CT extract concentration of 3% of DM. In the present study, the addition of PEG showed an increase in NH_3_-N and iso-butyrate and iso-valerate concentrations in the rumen in agreement with previous in vitro studies with rumen cattle fluid [52,53,54]. The increase in NH_3_-N concentration is attributed to the inhibition of microbial deaminase by tannins, thereby inducing high protein degradability. The lower concentration of iso-acids in the presence of tannins was attributed to the ability of tannins to bind proteins and the subsequent protection from ruminal deamination as iso-acids are derived from amino acids catabolism in the rumen [38,52,55]. Therefore, a reduction in protein degradation in the rumen will increase the quantity of protein digested in the small intestine [56]. Even if the presence of tannins in *Desmanthus* protected the proteins from degradation in the rumen, it did not seem to have an impact on total VFA, daily LW gain and DMI. The linear increase in DMI with increases in *Desmanthus* and CT from 0% to 1.1% in the diet suggests that the tannin levels in the current study were not toxic to ruminal microbes and did not have a negative impact on animal performance.

It seems that the low-quality basal diet with low CP and high fiber was the major reason for the low animal performance observed in the present study. The low-quality feed in the present study (8.2% CP in the Rhodes grass hay) was chosen to mimic the low-quality feed base in Northern Australia. Poppi et al. [57] reported a dietary CP concentration below 6% for about nine months of the year in Mitchell grass (*Astrebla* spp.), a native Australian species. They also reported a CP below 6% for about two months and a CP averaging 8% six months of the year in the introduced buffel grass (*Cenchrus ciliaris*) pastures.

### 4.4. Effect of Desmanthus Level on CH_4_ Emissions

The non-significant difference in CH_4_ emissions between the two *Desmanthus* cultivars corroborates the in vitro report of Vandermeulen et al. [8] where they found a similar CH_4_ production between JCU1 and JCU4 in March and October. In contrast, these authors reported a higher CH_4_ emission in August with JCU4 compared to JCU1, as did Durmic et al. [9] using in vitro techniques.

Methane production was highly correlated to DMI (R^2^ = 0.74) (Figure 2) as shown in other studies [58], therefore CH_4_ yield was chosen to better understand the mechanism behind CH_4_ reduction. Methane yield followed a linear increase pattern with an increase in *Desmanthus* percentage in the diet (Figure 3a). The coefficient of determination was higher when the CH_4_ yield was expressed as a function of *Desmanthus* DMI (Figure 3b). The observation that the addition of *Desmanthus* in the diet reduces CH_4_ emissions agrees with in vitro data by Vandermeulen et al. [8]. They showed a CH_4_ abatement of 21% and 26% after 72 h in vitro incubation with Brahman cattle rumen fluid for JCU1 and JCU4, respectively compared to Rhodes grass. Durmic et al. [9] revealed mixed results regarding the potential of *Desmanthus* to reduce CH_4_ emissions. In comparison to the average CH_4_ emissions from 23 tropical grasses, they showed that JCU1 generally reduced CH_4_, whereas JCU4 generally increased CH_4_ emissions.

Vandermeulen et al. [8] reported a negative correlation between CH_4_ production (mL/g OM) and TP, total tannins, and CT in *Desmanthus* and a highly significant correlation between HT and CH_4_ production in vitro. Previous studies also showed a reduction in CH_4_ in the presence of tannins in vitro and in vivo [11,59]. In the present study, CH_4_ yield as a function of the percentage of CT in the diet, followed a linear regression pattern (Figure 3c). However, no correlation was found between CH_4_ yield and percentage of TP in the diet (*p* = 0.22). These results are in discordance with the study conducted in vitro by Jayanegara et al. [60] using 17 polyphenol-containing plants where they reported a significantly negative relationship between CH_4_ production and TP and tannins, but not with CT. They concluded that TP and total tannins were good predictors of CH_4_ reduction potential. Tannins affect rumen microbial ecology and metabolism [61], but the mechanisms by which they affect methanogenesis are yet to be defined [11]. Some studies suggested that the greater the tannin molecular weight, the greater the CH_4_ reduction as the ability to bind methanogens would be higher [62,63]. Condensed tannins have a high molecular weight (1900 to 28,000 Da) compared to HT (500 to 3000 Da) [11]. Naumann et al. [10] did not find any correlation between the molecular weight of CT and CH_4_ emissions. Furthermore, Naumann et al. [64] reported no correlation between protein-precipitable phenolics or the amount of bound protein and the molecular weight of CT, although they showed a correlation between the CT, protein-precipitable phenolics and bound protein. Aboagye et al. [65] showed that gallic acids in HT had the potential to lower CH_4_ and nitrous oxide emissions in beef cattle without reducing feed digestibility due to their ability to bind and precipitate proteins [66].

To our knowledge, only one study [8] analyzed *D. illinoensis* and reported the molecular weight of CT (866 Da). Jayanegara et al. [67] suggested that the measure of biological activity of tannins is more accurate than measuring the concentration of tannins in the plant when it comes to studying the CH_4_ mitigation potential of tannins. Vandermeulen et al. [8] added that the verification must use Rubisco as the model protein because it represents the principal protein in fresh fodder at rumen pH 7 [68].

It should be noted that in the present study, the increase in tannin concentration was due to the increase in *Desmanthus* concentration in the diet and not due to an increase in tannin content in the cultivars. Vandermeulen et al. [8] reported similar or lower HT than CT in their study. Herein, the amount of PEG added in the diet (3.2 g PEG/g CT for the highest level of CT in the diet) was higher than the optimum concentration of PEG (1.2 g PEG/g tannin) suggested by Makkar et al. [69] to have a response in CH_4_ emission. However, in the present study, the addition of PEG had no impact on CH_4_ yield. This finding disagrees with previous in vitro and in vivo studies [52,54,70]. Bhatta et al. [52] found a 6.5% decrease in CH_4_ output in vitro when cattle rumen fluid was incubated with 25% tannin on a DM basis without PEG. Animut et al. [70] also showed an increase in CH_4_ production in vivo in goats ranging from 9.6 L/day to 19.0 L/day when a diet containing tannins at 15% of DM was not supplemented with PEG. However, another in vitro study [54] comparing 21 medicinal and aromatic plant leaves as antimethanogenic additives in bull feeds, showed mixed results. They found the highest CH_4_ increase with PEG addition (57%) with *Clerodendrum inerme* containing a low tannin concentration (0.03% DM CT as leucocyanidin equivalent and 2.4% DM HT) whereas the *Terminalia cordifolia* containing the highest concentration of tannins (1% DM CT as leucocyanidin equivalent and 25.2% DM HT) showed an increase in CH_4_ with PEG addition of only 7.2%. With respect to the tannin effect depending on the plants, it seems that the effect of *Desmanthus* tannins in CH_4_ may have been less than with other plants. It is possible that the anti-methanogenic effect observed in our trial was due to the response of the rumen to improve nitrogen availability.

The lack of correlation between the CP or NDF offered in the diet and CH_4_ yield (g/kg DMI) in the present study is in contradiction with previous studies that reported significant correlations between diet quality and CH_4_ emissions [71,72]. This observation would support the role of tannins in reducing CH_4_ emissions rather than the higher diet quality induced by an increase of *Desmanthus* in the diet.

### 4.5. Effect of Desmanthus Level on Rumen Metabolites

The increase in total VFA concentration with increasing levels of *Desmanthus* and thus tannins in the diet, was contrary to the effect observed in some studies in which a reduction in VFA was observed with the addition of tannins to the diet [8,67]. Similar results as in the present study were observed by Avila et al. [73] and Dickhoefer et al. [74]. They associated the increase in VFA to the reduction in water intake in treatments containing CT rather than an effect on carbohydrate degradation. The increase in rumen VFA may also be due to the increased supply of fermentable organic matter in the form of protein-N because the amino acids resulting from proteolysis can be deaminated and the carbon skeletons formed as a result can be fermented to VFA [45,75].

Methane and propionate are usually negatively correlated due to competition for hydrogen [52]. The formation of acetic and butyric acids induces the production of H_2_ and CO_2_, whereas propionic acid production requires a net uptake of H_2_ resulting in a decrease in methanogenesis [76]. The present study showed the opposite trend with an increase in acetate and a decrease in propionate as the level of *Desmanthus* in the diet increased. This might be due to the high concentration of NDF which stayed relatively constant, even with an increase in *Desmanthus* in the diet (76% DM). As dietary NDF increases, so does the molar proportion of acetate and subsequent decrease in the proportion of propionate [45].

## 5. Conclusions

*Desmanthus leptophyllus* (JCU1) and *Desmanthus bicornutus* (JCU4) showed that irrespective of cultivar, incremental supplementation with *Desmanthus* level in the diet induced a linear decrease in CH_4_ production and increase in VFA concentration. The ability of tannins in *Desmanthus* to reduce CH_4_ emissions with the addition of PEG in the diet was inconclusive albeit CH_4_ yield was negatively correlated with CT in the diet. Nevertheless, supplementation with PEG increased rumen NH_3_-N and iso-acid concentrations, suggesting an effective ability of tannins in *Desmanthus* to bind rumen proteins. It was also apparent in this study that increasing the *Desmanthus* level in the diet, increased the DMI without increasing the daily LW gain. The hypothesis that, on a low-quality diet, feeding tropical steers with incremental levels of *Desmanthus* will decrease CH_4_ emissions due to the presence of tannins without negatively affecting rumen metabolites and increase the animals’ LW gain is partly true. Therefore, it is concluded that *Desmanthus* has a potential to maintain the LW of the animals and reduce in vivo CH_4_ emissions by beef cattle in the drier parts of Northern Australia possibly due to a combination of rumen fermentation and tannin effects. These findings could contribute to the larger global effort of reducing the impact of climate change and greenhouse gas emission. However, further in vivo investigation is needed to better understand the mechanism behind the observed CH_4_ reduction associated with *Desmanthus* supplementation in the diet.

## Figures and Tables

**Table 1:** Chemical composition (means ± s.e.) of the Rhodes grass hay and the two cultivars of *Desmanthus* (JCU1 and JCU4).

Variable	Hay	JCU1	JCU4
Dry matter (%)	90.9 ± 0.247	54.1 ± 1.99	42.5 ± 1.39
Crude protein (% DM)	8.2 ± 0.162	11.0 ± 0.378	14.6 ± 0.727
Acid detergent fibre (% DM)	45.0 ± 0.170	46.3 ± 0.467	36.8 ± 0.912
Neutral detergent fibre (% DM)	76.2 ± 0.266	67.4 ± 0.410	58.3 ± 0.886
Metabolizable energy (MJ/kg DM) ^1^	6.4 ± 0.0212	6.5 ± 0.0711	7.3 ± 0.0893
Total phenolics (% DM as catechin equivalent)	0.34 ± 0.0271	1.7 ± 0.118	2.3 ± 0.187
Condensed tannins (% DM)	ND	3.5 ± 0.194	3.7 ± 0.301

**^1^** Estimated from in vitro true digestibility as DM digestibility × 0.172 − 1.707 [23], DM = dry matter, MJ = megajoules, ND = not detected.

**Table 2:** Nutritive value (means ± s.e.) of the treatments (JCU1 and JCU4) in the four diet levels (0, 15, 22 and 31% *Desmanthus* in the diet).

Variable	*Desmanthus* cv.	% *Desmanthus* Diet				Species *p*-Value
		0	15	22	31	
**Crude protein (% DM)**	JCU1	8.7 ± 0.155 ^a^	8.5 ± 0.0336 ^a^	9.9 ± 0.522 ^ac^	9.2 ± 0.523 ^a^	0.0077
	JCU4	8.6 ± 0.159 ^a^	8.8 ± 0.228 ^a^	11.5 ± 0.669 ^bc^	11.8 ± 0.351 ^b^	
**Acid detergent fibre (% DM)**	JCU1	47.1 ± 0.501 ^ab^	47.3 ± 0.615 ^ab^	46.8 ± 0.714 ^ab^	49.6 ± 0.594 ^a^	0.037
	JCU4	46.7 ± 0.602 ^b^	46.5 ± 0.707 ^b^	46.1 ± 0.741 ^b^	47.8 ± 0.424 ^ab^	
**Neutral detergent fibre (% DM)**	JCU1	76.6 ± 0.574 ^a^	76.9 ± 0.757 ^a^	73.5 ± 0.483 ^b^	74.9 ± 0.733 ^ab^	NS
	JCU4	76.1 ± 0.688 ^a^	77.1 ± 0.929 ^a^	75.7 ± 0.510 ^ab^	77.4 ± 0.576 ^a^	
**Metabolizable energy (MJ/kg DM) ^1^**	JCU1	6.2 ± 0.0365 ^a^	6.1 ± 0.0590 ^a^	6.3 ± 0.0722 ^a^	7.5 ± 1.01 ^a^	NS
	JCU4	6.2 ± 0.0460 ^a^	6.1 ± 0.0675 ^a^	6.3 ± 0.0530 ^a^	8.2 ± 1.34 ^a^	
**Condensed tannins (% DM)**	JCU1	0 ^a^	0.53 ± 0.00855 ^b^	1.1 ± 0.0148 ^c^	0.92 ± 0.117 ^c^	NS
	JCU4	0 ^a^	0.40 ± 0.0308 ^b^	1.1 ± 0.157 ^c^	0.87 ± 0.0207 ^c^	

^1^ Estimated from in vitro true digestibility as DM digestibility × 0.172 – 1.707 [19], DM = dry matter. Means between columns and species within the same variable without the same alphabetical characters (a, b, c) represent statistical differences (*p* < 0.05). Comparisons between species (JCU1 and JCU4) for each variable are declared NS, not significant when *p* > 0.05.

**Table 3:** Relationship between the dry matter intake (kg/day), DMI per kg LW (%), daily liveweight gain (kg), CH_4_ production (g/day), CH_4_ yield (g/kg DMI) and the percentage of *Desmanthus* DM in the diet (means ± s.e.).

Variables	% *Desmanthus* Diet				RMSE	Linear *p*-Value	R^2^
	0	15	22	31			
**Dry matter intake (kg/day)**	3.8 ± 0.189	3.6 ± 0.171	4.6 ± 0.285	4.7 ± 0.265	0.59	0.00013	0.49
**DMI/kg LW (%)**	1.3 ± 0.0451	1.2 ± 0.0497	1.5 ± 0.0759	1.6 ± 0.0637	1.89	0.0001	0.36
**Daily liveweight gain (kg)**	0.018 ± 0.181	0.12 ± 0.0700	0.29 ± 0.187	0.18 ± 0.0663	0.42	NS	0.033
**CH_4_ production (g/day)**	76.5 ± 2.74	68.7 ± 3.25	85.6 ± 4.80	81.9 ± 4.52	9.65	0.030	0.50
**CH_4_ yield (g/kg DMI)**	19.1 ± 0.504	19.2 ± 0.943	18.9 ± 0.445	17.5 ± 0.572	2.09	0.009	0.20

DMI = dry matter intake, LW = liveweight, CH_4_ = methane, RMSE = root mean square error. Means between the percentage of *Desmanthus* level in the diet for each variable are declared NS, not significant when *p* > 0.05.

**Table 4:** Relationship between the products of rumen fermentation and the percentage of *Desmanthus* (on dry matter basis) in the diet.

Variables	% *Desmanthus* Diet				RMSE	Linear *p*-Value	R^2^
	0	15	22	31			
Total VFA (mg/100dL)	49.8 ± 1.38	68.8 ± 3.44	55.0 ± 2.77	74.3 ± 4.41	11.25	0.0001	0.36
Acetate (molar %)	70.6 ± 0.344	74.4 ± 0.447	71.9 ± 0.279	73.9 ± 0.142	1.74	0.00044	0.19
Propionate (molar %)	19.4 ± 0.217	16.4 ± 0.317	18.0 ± 0.193	16.5 ± 0.112	1.19	0.0001	0.30
Acetate/Propionate ratio	3.7 ± 0.0592	4.6 ± 0.124	4.0 ± 0.0586	4.5 ± 0.0374	0.40	0.00011	0.22
Iso-butyrate (molar %)	0.52 ± 0.0248	0.50 ± 0.0311	0.44 ± 0.0355	0.43 ± 0.0209	0.090	0.012	0.21
n-butyrate (molar %)	8.2 ± 0.19	7.3 ± 0.218	8.3 ± 0.136	7.7 ± 0.0943	0.70	NS	0.029
Iso-valerate (molar %)	0.66 ± 0.0469	0.64 ± 0.0481	0.51 ± 0.0525	0.58 ± 0.0418	0.13	0.039	0.32
n-valerate (molar %)	0.52 ± 0.0177	0.68 ± 0.0254	0.71 ± 0.0408	0.74 ± 0.0287	0.082	0.0001	0.51
n-caproate (molar %)	0.13 ± 0.00833	0.11 ± 0.0129	0.18 ± 0.00690	0.13 ± 0.00732	0.036	NS	0.13
NH_3_-N (mg/dL)	6.4 ± 0.550	6.6 ± 0.410	7.7 ± 1.29	8.0 ± 0.489	2.77	0.033	0.16
pH	7.0 ± 0.0607	6.9 ± 0.0583	7.1 ± 0.0696	6.9 ± 0.0470	0.20	NS	0.15

VFA = volatile fatty acids. Values are means ± s.e., RMSE = root mean square error. Means between the *Desmanthus* level in the diet for each variable are declared NS, not significant when *p* > 0.05.
